# Impact of Comorbidities on Survival in Gastric, Colorectal, and Lung Cancer Patients

**DOI:** 10.2188/jea.JE20170241

**Published:** 2019-03-05

**Authors:** Toshitaka Morishima, Yoshifumi Matsumoto, Nobuyuki Koeda, Hiroko Shimada, Tsutomu Maruhama, Daisaku Matsuki, Kayo Nakata, Yuri Ito, Takahiro Tabuchi, Isao Miyashiro

**Affiliations:** 1 Cancer Control Center, Osaka International Cancer Institute, Osaka, Japan; 2 Yao Municipal Hospital, Osaka, Japan; 3 National Hospital Organization Osaka Minami Medical Center, Osaka, Japan; 4 Higashisumiyoshi Morimoto Hospital, Osaka, Japan; 5 Saiseikai Suita Hospital, Osaka, Japan

**Keywords:** administrative claims data, comorbidity, medical record linkage, neoplasms, survival analysis

## Abstract

**Background:**

The presence of comorbidities in cancer patients may influence treatment decisions and prognoses. This study aimed to examine the impact of comorbidities on overall survival in Japanese patients diagnosed with major solid tumors.

**Methods:**

To obtain patient-level information on clinical conditions and vital status, we performed a record linkage of population-based cancer registry data from Osaka Prefecture, Japan and administrative data produced under the Diagnosis Procedure Combination (DPC) system. The study population comprised patients who received a primary diagnosis of gastric, colorectal, or lung cancer between 2010 and 2012 at any of five cancer centers. We employed the Charlson Comorbidity Index (CCI) score to quantify the impact of comorbidities on survival. The association between CCI score and survival for each cancer site was analyzed using Cox proportional hazards regression models for all-cause mortality, after adjusting for patient sex, age at cancer diagnosis, and cancer stage.

**Results:**

A total of 2,609 patients with a median follow-up duration of 1,372 days were analyzed. The most frequent CCI score among the patients was 0 (77.7%), followed by 2 (14.3%). After adjusting for the covariates, we detected a significant association between CCI score and all-cause mortality. The hazard ratios per one-point increase in CCI score were 1.12 (95% confidence interval [CI], 1.02–1.23), 1.20 (95% CI, 1.08–1.34), and 1.14 (95% CI, 1.04–1.24) for gastric, colorectal, and lung cancer, respectively.

**Conclusions:**

Comorbidities have a negative prognostic impact on overall survival in cancer patients, and should be assessed as risk factors for mortality when reporting outcomes.

## INTRODUCTION

Numerous patients with cancer also present with one or more comorbidities, which refer to co-occurring non-cancer conditions that are distinct from the principal diagnosis. Because the incidences of chronic diseases and cancer increase with age, the prevalence of comorbidities tends to be higher in older cancer patients.^1^^,^^2^ Even if cancer incidence rates remain stable or decline, the number of older cancer patients with comorbidities is expected to rise due to population aging and advances in treatments.^3^ Comorbidities present considerable challenges to cancer management because of their potential impact on treatment decisions.^4^^,^^5^ In addition, comorbid conditions may also independently increase a patient’s risk of death.^6^ Examining regional or institutional disparities in outcomes generally involves adjusting for the inherent differences in patient case mix.^7^^,^^8^ Accordingly, gaining a better understanding of the relationship between comorbidities and cancer survival may contribute to fairer assessments of a patient’s prognosis and quality of care.

Although population-based cancer registries do not routinely collect data on non-cancer conditions, this information is commonly available in administrative claims data. Therefore, the linkage of these two data sources represents a possible strategy to provide more accurate survival estimates based on both cancer characteristics and comorbidities.^9^ Several studies have previously demonstrated the impact of comorbidities on cancer survival, and some have used record linkages of registry data and administrative data.^1^^,^^10^^–^^17^ While these studies have reported that comorbidities can affect cancer survival, they contained several methodological limitations, such as focusing only on elderly patients, single-site cancers, single-institution data, and small sample sizes.^18^^,^^19^ Furthermore, few studies have investigated the impact of comorbidities on survival in gastric cancer patients.^10^^,^^11^ This is because previous studies were generally performed using data from the United States and Europe, where gastric cancer is less prevalent. In contrast, over 50% of all gastric cancer cases are diagnosed in East Asia.^20^

This multi-center study aimed to examine the impact of comorbidities on overall survival in patients diagnosed with gastric, colorectal, or lung cancer using cancer registry data linked with administrative data.

## METHODS

### Data sources

In this study, we linked two data sources to obtain a large consolidated database in order to analyze the relationship between patient mortality and clinical information that is not routinely collected in a cancer registry. Data from 2010 through 2012 were obtained for analysis.

The first data source was the Osaka Cancer Registry (OCR), which is a population-based cancer registry that collects information on cancer diagnoses and outcomes in residents of Osaka Prefecture, Japan. Patient data from the OCR include sex, age at cancer diagnosis, vital status, and dates of death or the last follow-up for vital status. Tumor-specific data include cancer site; Surveillance, Epidemiology, and End Results (SEER) summary stage at diagnosis^21^; and date of cancer diagnosis. We chose to use population-based registry data instead of hospital-based registry data because the former actively works to track the vital status of all registered cancer patients. As a result, population-based registry data would likely provide a higher follow-up rate than hospital-based registry data. Follow-up for vital status are routinely performed using death certificates. In addition, patients diagnosed with cancer in 2010 and 2012 were followed up in December 2016 using official resident registries to verify vital status.

The second data source was administrative data produced under Japan’s Diagnosis Procedure Combination (DPC) Per-Diem Payment System, which prescribes reimbursements from insurers to acute care hospitals. DPC data represent one of the most widely used hospital administrative data sources for research in Japan.^22^ Clinical summaries and detailed insurance claims can be extracted from these data. We collected DPC data from five hospitals that are designated as cancer centers by the national or prefectural government. From 2010 through 2012, these five hospitals treated 8.6%, 7.4%, and 7.1% of all gastric, colorectal, and lung cancer patients, respectively, within the prefecture.

The two sources of data were linked at the patient level using each hospital’s patient identification number as the linkage key. Approximately 95% of the patients in the OCR database who had been diagnosed with cancer at the five hospitals were matched with their corresponding DPC data.

### Study population

Cancer was identified according to the topographical codes of the International Classification of Diseases for Oncology, Third Edition. We initially selected patients who had been diagnosed with gastric (C16.x), colorectal (C18.x, C19.x, C20.x), or lung (C33.x, C34.x) cancer at the age of 18 years or older at the five subject hospitals and had been included in the OCR between January 1, 2010 and December 31, 2012. These three cancer sites were chosen because they are the most common cancers that occur in the prefecture and accounted for 44% of all new cancer cases in 2012.^23^ Patients were excluded if they had a missing vital status or a diagnosis of carcinoma in situ.

### Measurement of comorbidities

The format of DPC data allows hospitals to report the presence of comorbidities in each patient. All comorbidities that were present at the time of admission were identified from the relevant data fields in the first inpatient DPC file during the period from 3 months before to 3 months after cancer diagnosis. This long period provided a substantial duration for the diagnosis of cancer and its associated comorbidities. A maximum of four comorbidities can be recorded in DPC data fields that are separate from the main diagnosis and complications. These comorbidities are recorded using International Classification of Diseases, Tenth Revision (ICD-10) codes.

The comorbidity measure used in this study included the following 16 comorbid conditions identified by Charlson et al^24^: myocardial infarction, congestive heart failure, peripheral vascular disease, cerebrovascular disease, dementia, chronic pulmonary disease, connective tissue disease, peptic ulcer, mild liver disease, moderate or severe liver disease, diabetes without chronic complications, diabetes with chronic complications, hemiplegia or paraplegia, renal disease, hematological and solid cancer diagnosed before the cancer of interest, and acquired immunodeficiency syndrome (AIDS) or human immunodeficiency virus (HIV). Although the Charlson Comorbidity Index (CCI) typically includes metastatic cancer diagnoses, this condition was excluded because it may be an extension of the cancer of interest.^12^^–^^14^ Also, a cancer diagnosis as a comorbidity that was the same as the cancer of interest was excluded. For example, a diagnosis of gastric cancer as a comorbidity was excluded when calculating the CCI in gastric cancer patients. The presence or absence of the 16 comorbidities was determined according to an algorithm developed by Quan et al for ICD-10 codes recorded in administrative data.^25^

The CCI was used as a summary measure of comorbidities, in which each comorbid condition was assigned a weight based on its association with mortality; the weights were then summed to calculate the CCI for each patient. In 2011, Quan et al released new weights for the CCI, and five conditions were assigned a weight of 0 to account for advances in healthcare technology (the updated weights are provided in Table 1).^26^ The revised CCI score used in this study was therefore calculated by summing the weighted score of 11 conditions. During the calculation of the CCI score, double counting of overlapping categories was avoided. For example, if a patient suffered from asthma and chronic obstructive pulmonary disease, this constituted only one point as both conditions are from the same category (ie, chronic pulmonary disease).

**Table 1.** Patient characteristics and prevalence of comorbidities

	Gastric cancer	Colorectal cancer	Lung cancer	Weighted score in CCI
Total	1,022 (100)	791 (100)	796 (100)	
All-cause mortality	426 (42)	311 (39)	517 (65)	
Median follow-up duration, days (IQR)	1,460 (451–1,681)	1,520 (708–1,724)	621 (256–1,531)	
Male	706 (69)	420 (53)	542 (68)	
Age at diagnosis				
<65 years	279 (27)	236 (30)	214 (27)	
65–69 years	189 (18)	141 (18)	161 (20)	
70–74 years	205 (20)	133 (17)	160 (20)	
75–79 years	202 (20)	134 (17)	142 (18)	
≥80 years	147 (14)	147 (19)	119 (15)	
Stage at diagnosis				
Localized	550 (54)	332 (42)	250 (31)	
Regional to lymph nodes	118 (12)	158 (20)	103 (13)	
Regional by direct extension	100 (10)	88 (11)	102 (13)	
Distant	246 (24)	189 (24)	318 (40)	
Unknown	8 (1)	24 (3)	23 (3)	
Comorbidities				
Myocardial infarction	5 (0)	8 (1)	10 (1)	0
Congestive heart failure	22 (2)	14 (2)	27 (3)	2
Peripheral vascular disease	20 (2)	11 (1)	13 (2)	0
Cerebrovascular disease	37 (4)	20 (3)	39 (5)	0
Dementia	7 (1)	12 (2)	7 (1)	2
Chronic pulmonary disease	33 (3)	19 (2)	59 (7)	1
Connective tissue disease	2 (0)	2 (0)	2 (0)	1
Peptic ulcer	266 (26)	75 (9)	35 (4)	0
Mild liver disease	51 (5)	42 (5)	43 (5)	2
Moderate or severe liver disease	4 (0)	1 (0)	1 (0)	4
DM without chronic complications	106 (10)	72 (9)	84 (11)	0
DM with chronic complications	11 (1)	22 (3)	19 (2)	1
Hemiplegia or paraplegia	1 (0)	1 (0)	0 (0)	2
Renal disease	13 (1)	7 (1)	3 (0)	1
Other cancer	79 (8)	59 (7)	73 (9)	2
AIDS or HIV	0 (0)	0 (0)	0 (0)	4

AIDS, acquired immunodeficiency syndrome; DM, diabetes mellitus; HIV, human immunodeficiency virus; IQR, interquartile range.

Values are expressed as number (column percentage) unless otherwise indicated. Because of rounding, percentages may not add up to 100%.

### Statistical analyses

The outcome of interest was all-cause mortality. Duration of survival was measured from the date of cancer diagnosis until the date of death or the censor date of the last follow-up for vital status. Multivariable Cox proportional hazards regression analysis was performed to adjust for patient and tumor characteristics.^11^^–^^17^ Two models were developed for each cancer site. The first model was a “partial” model that controlled for sex, age at cancer diagnosis (<65 years, 65–69 years, 70–74 years, 75–79 years, and ≥80 years), and cancer stage at diagnosis (localized, regional to lymph nodes, regional by direct extension, distant, or unknown). The second model was a “full” model that, in addition to the covariates in the partial model, also included the CCI score as a continuous variable.^15^^,^^16^^,^^27^ The CCI score was analyzed as a continuous variable because it was validated as such in the study by Quan et al.^26^ Adjusted hazard ratios (HRs) and 95% confidence intervals (CIs) for all-cause mortality of the covariates and CCI score were calculated. Violation of the proportional hazards assumption was not observed upon inspection of the log-log survival curve plots (data not shown).^28^

To assess the incremental prognostic value of the CCI score, we estimated Harrell’s concordance statistic (C-statistic) of the two models for each cancer site.^29^ This statistic is comparable to the area under the receiver operating characteristic curve, where a value of 0.5 indicates random predictions and a value of 1.0 indicates perfect discrimination between survivors and non-survivors. In addition, goodness of fit for sequential models was compared using the Akaike information criterion (AIC), where a smaller AIC value indicates a more desirable model for predicting outcomes.

Furthermore, relative survival stratified by the CCI score (0 and ≥1) was analyzed. The outcome measure was the ratio of observed to expected survival derived from the general Japanese population that was similar to the cancer patients in terms of sex and age based on life tables for all-cause mortality using the Ederer II method.^30^^,^^31^ We also estimated the excess hazard ratios (EHRs) of all-cause mortality for sex, age, cancer stage, and CCI score using excess hazard modelling.^32^ Cox proportional hazards regression analyses were performed using SAS version 9.4 (SAS Institute, Cary, NC, USA), and relative survival and EHRs were estimated using Stata version 15.1 (StataCorp LP, College Station, TX, USA). All statistical tests were two-sided, and a *P* value of less than 0.05 was considered statistically significant. The present study was approved by the Institutional Review Board of Osaka International Cancer Institute (Approval number: 1607289079).

## RESULTS

### Study population

A total of 2,609 patients were included in the analysis. There were 1,022 gastric cancer patients, 791 colorectal cancer patients, and 796 lung cancer patients. The patient characteristics are summarized in Table 1. At the time of OCR data acquisition (March 2017), 42%, 39%, and 65% of gastric, colorectal, and lung cancer patients, respectively, had died. The median duration of follow-up for the study population was 1,372 days; the median durations were approximately 1,500 days for gastric and colorectal cancer patients and 621 days for lung cancer patients. Among the gastric and colorectal cancer patients, the “localized” stage was the most common cancer stage according to SEER criteria; the “distant” stage was the most common cancer stage in lung cancer patients.

Table 1 also shows the prevalence of the 16 comorbidities according to cancer site. The most prevalent conditions were peptic ulcer (gastric and colorectal cancer patients) and diabetes without chronic complications (lung cancer patients). No patients with AIDS or HIV were identified. The distribution of the CCI score was 0 in 2,026 patients (78%), 1 in 139 (5%), 2 in 374 (14%), 3 in 20 (1%), and ≥4 in 50 (2%) (Table 2). A large proportion of gastric cancer patients (80%), colorectal cancer patients (80%), and lung cancer patients (73%) had a CCI score of 0.

**Table 2.** Distribution of the Charlson Comorbidity Index score

	Gastric cancer	Colorectal cancer	Lung cancer
Total	1,022 (100)	791 (100)	796 (100)
CCI score			
0	817 (80)	631 (80)	578 (73)
1	40 (4)	36 (5)	63 (8)
2	142 (14)	102 (13)	130 (16)
3	5 (0)	7 (1)	8 (1)
≥4	18 (2)	15 (2)	17 (2)

CCI, Charlson Comorbidity Index.

Values are expressed as number (column percentage) unless otherwise indicated. Because of rounding, percentages may not add up to 100%.

### Prognostic impact of comorbidities

Table 3 presents the adjusted HRs for all-cause mortality of the covariates and CCI score at diagnosis calculated using Cox proportional hazards regression analyses. Sex, age, and cancer stage yielded similar HRs in both the partial and full models. In all three cancer sites, increasing age and cancer stage progression were significantly associated with higher HRs for all-cause mortality.

**Table 3.** Adjusted hazard ratios of all-cause mortality derived from Cox proportional hazards models according to cancer type

	Gastric cancer				Colorectal cancer				Lung cancer			
		
	Partial model		Full model		Partial model		Full model		Partial model		Full model	
	HR (95% CI)	*P* value	HR (95% CI)	*P* value	HR (95% CI)	*P* value	HR (95% CI)	*P* value	HR (95% CI)	*P* value	HR (95% CI)	*P* value
Sex (Ref = female)												
Male	1.22 (0.98–1.51)	0.073	1.21 (0.97–1.49)	0.090	0.99 (0.79–1.24)	0.90	1.00 (0.79–1.25)	0.97	1.63 (1.34–2.00)	<0.001	1.57 (1.28–1.92)	<0.001
Age (Ref = <65 years)												
65–69 years	1.44 (1.06–1.95)	0.020	1.45 (1.07–1.97)	0.017	1.02 (0.72–1.45)	0.91	1.06 (0.75–1.52)	0.73	1.29 (0.98–1.69)	0.066	1.25 (0.96–1.65)	0.103
70–74 years	1.53 (1.13–2.08)	0.007	1.54 (1.13–2.09)	0.006	1.45 (1.01–2.09)	0.046	1.38 (0.96–1.99)	0.086	1.82 (1.39–2.39)	<0.001	1.76 (1.34–2.32)	<0.001
75–79 years	1.93 (1.42–2.62)	<0.001	1.91 (1.41–2.60)	<0.001	1.69 (1.20–2.38)	0.003	1.72 (1.22–2.42)	0.002	2.53 (1.93–3.32)	<0.001	2.43 (1.85–3.19)	<0.001
≥80 years	3.82 (2.83–5.14)	<0.001	3.74 (2.77–5.04)	<0.001	3.27 (2.35–4.56)	<0.001	3.17 (2.27–4.43)	<0.001	3.81 (2.87–5.07)	<0.001	3.67 (2.76–4.89)	<0.001
Stage at diagnosis (Ref = localized)												
Regional to lymph nodes	5.01 (3.46–7.25)	<0.001	5.03 (3.47–7.28)	<0.001	2.33 (1.56–3.49)	<0.001	2.30 (1.54–3.43)	<0.001	5.28 (3.69–7.53)	<0.001	5.45 (3.81–7.79)	<0.001
Regional by direct extension	10.60 (7.52–14.95)	<0.001	10.70 (7.59–15.09)	<0.001	4.42 (2.95–6.64)	<0.001	4.51 (3.00–6.77)	<0.001	6.45 (4.53–9.19)	<0.001	6.60 (4.63–9.40)	<0.001
Distant	33.89 (25.15–45.67)	<0.001	34.17 (25.35–46.06)	<0.001	14.97 (10.67–20.99)	<0.001	14.96 (10.66–20.98)	<0.001	13.13 (9.73–17.72)	<0.001	13.61 (10.07–18.39)	<0.001
Unknown	54.32 (24.31–121.40)	<0.001	56.72 (25.36–126.89)	<0.001	5.99 (3.35–10.72)	<0.001	5.32 (2.96–9.57)	<0.001	9.73 (5.79–16.38)	<0.001	10.11 (6.00–17.02)	<0.001
CCI score			1.12 (1.02–1.23)^a^	0.020			1.20 (1.08–1.34)^a^	0.001			1.14 (1.04–1.24)^a^	0.006

CCI, Charlson Comorbidity Index; CI, confidence interval; HR, hazard ratio; Ref, reference.

All models include the baseline variables of sex, age at diagnosis, and cancer stage at diagnosis. Full models also include the CCI score in addition to the baseline variables.

^a^Expressed as the hazard ratio per one-point increase in CCI score.

To assess the prognostic impact of comorbidities after controlling for the other covariates, the full model (in which the CCI score was entered on top of the partial model’s covariates) was constructed for each of the three cancer sites. The adjusted HRs for all-cause mortality for a single-point elevation in the CCI score were 1.12 (95% CI, 1.02–1.23), 1.20 (95% CI, 1.08–1.34), and 1.14 (95% CI, 1.04–1.24) for gastric, colorectal, and lung cancer patients, respectively.

### Cox proportional hazards regression model performance

To estimate the incremental prognostic value of the CCI score and the goodness of fit in the different models, we compared the C-statistic and AIC values of the partial model and full model for each of the three cancer sites (Table 4). When compared with the partial model, the full model with the CCI score yielded slightly higher C-statistic and lower AIC values for all three cancer sites. The addition of the CCI score provided little improvement to the discriminatory power of the prognostic models.

**Table 4.** Assessment of Cox proportional hazards models with all-cause mortality as the dependent variable

	Gastric cancer		Colorectal cancer		Lung cancer	
		
	Partial model	Full model	Partial model	Full model	Partial model	Full model
C-statistic	0.856	0.859	0.784	0.789	0.787	0.789
AIC	4,887	4,884	3,643	3,636	5,913	5,908

AIC, Akaike Information Criterion; C-statistic, Harrell’s concordance statistic.

All models include the baseline variables of sex, age at diagnosis, and cancer stage at diagnosis. Full models also include the Charlson Comorbidity Index score in addition to the baseline variables.

### Relative survival and excess hazard ratios

Relative survival and adjusted EHRs are presented in the supplementary tables. As shown in eTable 1, the 3-year relative survival of gastric, colorectal, and lung cancer patients with a CCI score of ≥1 was lower than those with a CCI score of 0 (61.4% vs 69.1%, 63.2% vs 75.3%, and 38.1% vs 44.7%, respectively). In addition, we observed a significantly higher risk of adjusted excess deaths that occurred in colorectal and lung cancer patients with comorbidities (eTable 2). The adjusted EHRs of all-cause mortality for a single-point elevation in the CCI score were 1.08 (95% CI, 0.96–1.22), 1.18 (95% CI, 1.02–1.37), and 1.13 (95% CI, 1.02–1.25) for gastric, colorectal, and lung cancer patients, respectively. These EHRs were similar to the HRs calculated from the Cox proportional hazards regression analyses.

## DISCUSSION

The present study highlights the impact of comorbidities on overall survival in patients diagnosed with gastric, colorectal, or lung cancer. Data on individual comorbid conditions from administrative data were combined with survival status from population-based registry data, and this combination of data sources is increasingly employed for research purposes to improve analytic efficiency with fewer variables.^9^^,^^19^ We found that the presence of comorbidities was significantly associated with elevated all-cause mortality in patients diagnosed with any of the three target cancers, even after adjusting for sex, age, and cancer stage. Our results therefore indicated that information on comorbidities was prognostically relevant in these patients. The prognostic impact of comorbidities may be due to their relatively direct effect on survival.^6^ In addition, increasingly severe comorbidities may also be associated with increased toxicity of specific treatments or the use of less optimal or aggressive therapy, thereby reducing a patient’s remaining life expectancy.^4^^,^^5^

The difference in HRs for each comorbidity among the gastric, colorectal, and lung cancer patients may be due to the varying aggressiveness of the cancer types. The effect of comorbidities on mortality may be relatively small for cancers with generally poor prognosis.^17^ Patients with rapidly growing cancer are more likely to die from the cancer than from their comorbidities, whereas patients with slowly-growing cancer are more susceptible to other conditions.^11^

The predictive abilities of the models used in this study were evaluated using the C-statistic.^29^ When compared with the partial model, the addition of the CCI score to the full model resulted in a better goodness of fit, but increased the C-statistic by only 0.002 to 0.005. This is consistent with the results of a previous study, where the addition of comorbidities led to increases in the C-statistic that ranged from 0.00 for lung cancer to 0.04 for prostate cancer.^17^

The low incremental prognostic value of comorbidities in the present study may be due to several reasons. First, the relatively poor ability of the CCI score derived from Japanese administrative data to predict 1-year survival has been previously demonstrated in an international comparison of databases.^26^ This may have resulted from a lack of financial incentives that ensure the complete reporting of comorbidities by hospitals, as well as the limited number of comorbidity fields in DPC data.^33^ Second, it has been reported that the value of the CCI score derived from Japanese administrative data tends to be lower than the corresponding score derived from chart review, despite the high specificity of the comorbid conditions in the former.^33^ Third, the application of CCI may be suboptimal for cancer patients. Klabunde et al developed the National Cancer Institute comorbidity index, which utilizes a comorbidity measurement algorithm that is optimized for studying common cancers.^34^ Although it has been adapted for use with ICD-9 diagnostic and procedural codes, the development of a cancer-specific comorbidity index based on ICD-10 codes may be beneficial. Fourth, the impact of comorbidities on survival may be attenuated when the CCI score was entered on top of the partial model’s covariates because the effects of comorbidities had already been controlled in part by the partial model (including patient age).

### Limitations

Our study has several potential limitations. First, we extracted comorbidity information from an administrative data source that does not provide as complete or detailed an identification of comorbidities as clinical databases. However, most studies that have examined the impact of comorbidities on cancer survival were based on analyses of administrative data linked with cancer registry data.^19^

Second, the measurement of comorbidities did not take into account disease severity or how long a patient has had each condition. This was because the majority of comorbidity codes in administrative data are provided as dichotomous variables. None of the earlier studies have examined the impact of duration and/or severity of comorbidities on cancer survival.^18^

Third, coding practice may vary over time and among institutions. This could limit the generalizability of results and potentially influence the relationships between comorbidity and overall survival. However, there are currently no data to clarify the variations in coding practice.

Fourth, there may be a selection bias toward hospitalized patients with relatively mild comorbid conditions because the study population was recruited from designated cancer centers that tend to target patients with fewer comorbidities. In contrast, other types of acute care hospitals and long-term care facilities may treat patients with more comorbidities and poorer prognosis. This bias may consequently weaken the signal of the association between comorbidities and cancer survival, resulting in an underestimation of the impact of comorbidities. It is therefore possible that the inclusion of data from other types of institutions would have resulted in higher HRs than those reported here.

Fifth, we had excluded metastatic cancer diagnoses when calculating the CCI score for each patient in order to avoid over-adjustment. However, the condition of metastatic solid tumors has a weighted score of 6 in the CCI, and the exclusion of patients with these tumors may therefore underestimate the impact of comorbidities on survival.

### Implications

There is an increasing recognition of the need to measure comorbidity in studies that compare outcomes among different providers or regions in order to remove factors that may influence observed differences in outcomes.^7^^,^^8^ Although the methodology of risk adjustment is continuously refined, a degree of risk adjustment is needed to avoid unfairly damaging the reputation of providers that treat high-risk or complex patients.^35^ In addition, improved descriptions of patients with cancer may result in improved prognostic stratification, thereby allowing more accurate estimates of treatment effectiveness when conducting outcomes research and analyzing data from cancer registries.^4^^,^^5^^,^^17^

### Conclusions

The findings of the present study underscore the importance of including comorbid conditions in the prognostic assessment of cancer patients. The addition of information on comorbidities derived from administrative data may allow for more accurate risk adjustments than the use of cancer registry data alone.

## APPENDIX A. SUPPLEMENTARY DATA

eTable 1. Observed and relative survival according to cancer type

eTable 2. Adjusted excess hazard ratios of all-cause mortality derived from excess hazard models according to cancer type
