# Supplementary material for: Impact of Comorbidities on Survival in Gastric, Colorectal, and Lung Cancer Patients
Source: J Epidemiol. 2019 Mar 5;29(3):110–5. doi: 10.2188/jea.JE20170241 (PMC6375811; doi:10.2188/jea.JE20170241)
Supplement: Supplementary file 1 [file je-29-110-s001.pdf]

eTable 1. Observed and relative survival according to cancer type

			3-year observed survival, Percentage (95% CI)		3-year relative survival, Percentage (95% CI)	
			CCI=0	CCI ≥1	CCI=0	CCI ≥1
Gastric cancer	All		64.3 (60.9–67.5)	56.0 (48.9–62.5)	69.1 (65.4–72.5)	61.4 (53.6–68.5)
	Sex	Female	67.6 (61.5–73.0)	57.9 (44.1–69.5)	71.3 (64.9–77.0)	61.0 (46.4–73.2)
		Male	62.8 (58.6–66.7)	55.2 (46.8–62.8)	68.1 (63.6–72.3)	61.5 (52.1–70.0)
	Age at diagnosis	<65 years	70.7 (64.5–76.1)	65.0 (48.2–77.6)	72.1 (65.7–77.6)	66.3 (49.1–79.1)
		65–69 years	46.9 (37.3–56.0)	41.0 (25.7–55.8)	58.7 (46.6–70.1)	52.3 (32.7–71.0)
		70–74 years	62.8 (54.8–69.8)	48.3 (29.5–64.8)	65.3 (57.0–72.6)	50.4 (30.7–67.6)
		75–79 years	66.0 (58.0–72.8)	62.0 (46.1–74.4)	70.3 (61.8–77.6)	66.2 (49.3–79.4)
		≥80 years	66.6 (58.4–73.5)	59.3 (44.7–71.3)	74.6 (65.5–82.4)	66.6 (50.1–80.0)
	Stage at diagnosis	Localized	95.4 (92.9–97.0)	84.2 (75.8–89.8)	102.6 (100.0–104.3)	92.6 (83.4–98.8)
		Regional to lymph nodes	65.2 (54.3–74.1)	51.7 (32.5–67.9)	69.4 (57.8–78.9)	57.0 (35.8–74.8)
		Regional by direct extension	36.4 (26.0–46.9)	36.8 (16.5–57.5)	40.0 (28.5–51.4)	39.4 (17.7–61.5)
		Distant	8.2 (4.9–12.6)	3.0 (0.3–12.4)	8.7 (5.2–13.3)	3.2 (0.3–13.1)
		Unknown	16.7 (0.8–51.7)	NA	16.9 (0.8–52.4)	NA
Colorectal cancer	All		70.1 (66.3–73.5)	57.3 (49.2–64.6)	75.3 (71.3–79.0)	63.2 (54.3–71.2)
	Sex	Female	69.2 (63.7–74.1)	56.0 (43.5–66.9)	73.4 (67.5–78.6)	60.6 (47.0–72.2)
		Male	70.8 (65.6–75.5)	58.2 (47.4–67.6)	77.1 (71.3–82.1)	65.3 (53.2–75.8)
	Age at diagnosis	<65 years	72.9 (66.2–78.5)	74.4 (55.3–86.3)	74.1 (67.3–79.8)	76.1 (56.6–88.3)
		65–69 years	64.1 (53.8–72.6)	47.8 (32.9–61.3)	81.5 (68.5–92.4)	59.8 (41.2–76.6)
		70–74 years	70.7 (61.8–77.9)	68.8 (40.5–85.6)	73.3 (64.1–80.8)	71.6 (42.1–89.2)
		75–79 years	77.2 (67.5–84.4)	50.0 (32.9–64.9)	81.7 (71.4–89.3)	53.0 (34.9–68.8)
		≥80 years	63.1 (53.0–71.6)	56.7 (37.3–72.1)	69.5 (58.4–78.9)	62.8 (41.3–79.8)
	Stage at diagnosis	Localized	91.5 (87.5–94.3)	86.1 (74.1–92.8)	98.6 (94.3–101.6)	97.6 (84.0–105.2)
		Regional to lymph nodes	82.5 (74.4–88.2)	60.0 (42.0–74.0)	89.1 (80.3–95.3)	65.3 (45.7–80.6)
		Regional by direct extension	64.7 (52.4–74.6)	41.2 (18.6–62.6)	70.1 (56.8–80.9)	44.2 (19.9–67.2)
		Distant	25.2 (18.5–32.4)	25.0 (13.0–39.0)	26.5 (19.5–34.0)	26.2 (13.6–41.0)
		Unknown	50.0 (22.9–72.2)	40.0 (12.3–67.0)	53.5 (24.5–77.3)	45.1 (13.8–75.6)

eTable 1. continued

			3-year observed survival, Percentage (95% CI)		3-year relative survival, Percentage (95% CI)	
			CCI=0	CCI ≥1	CCI=0	CCI ≥1
Lung cancer	All		42.1 (38.0–46.1)	35.3 (29.0–41.8)	44.7 (40.4–49.0)	38.1 (31.3–45.1)
	Sex	Female	54.3 (47.2–60.9)	46.9 (32.8–59.9)	56.4 (49.0–63.3)	48.4 (33.8–61.7)
		Male	35.5 (30.6–40.3)	31.8 (24.8–39.0)	38.3 (33.0–43.5)	34.9 (27.3–42.9)
	Age at diagnosis	<65 years	50.6 (43.0–57.7)	68.8 (50.6–81.4)	51.6 (43.8–58.8)	70.1 (51.6–82.9)
		65–69 years	28.2 (18.8–38.3)	13.5 (4.9–26.4)	34.4 (23.0–46.8)	18.0 (6.6–35.2)
		70–74 years	45.8 (36.5–54.6)	38.3 (24.4–52.1)	47.6 (37.9–56.7)	40.0 (25.5–54.5)
		75–79 years	40.4 (31.2–49.5)	40.0 (26.5–53.1)	42.9 (33.1–52.5)	42.7 (28.3–56.7)
		≥80 years	35.5 (25.9–45.3)	19.6 (9.7–32.0)	39.4 (28.7–50.2)	22.1 (11.0–36.0)
	Stage at diagnosis	Localized	89.1 (83.4–92.9)	72.3 (60.1–81.4)	94.9 (88.8–98.9)	78.2 (64.9–88.0)
		Regional to lymph nodes	38.4 (27.3–49.3)	33.3 (17.5–50.0)	40.8 (29.1–52.5)	35.7 (18.8–53.6)
		Regional by direct extension	34.9 (24.1–45.9)	17.9 (6.5–33.7)	36.9 (25.5–48.6)	19.5 (7.1–36.8)
		Distant	11.5 (7.8–15.9)	12.4 (6.2–21.0)	12.1 (8.2–16.7)	13.2 (6.6–22.3)
		Unknown	33.3 (10.3–58.8)	9.1 (0.5–33.3)	36.9 (11.4–65.2)	10.0 (0.6–36.4)

CCI, Charlson Comorbidity Index; CI, confidence interval; NA, not applicable.

eTable 2. Adjusted excess hazard ratios of all-cause mortality derived from excess hazard models according to cancer type

	Gastric cancer				Colorectal cancer				Lung cancer			
	Partial model		Full model		Partial model		Full model		Partial model		Full model	
	EHR (95% CI)	*P* value	EHR (95% CI)	*P* value	EHR (95% CI)	*P* value	EHR (95% CI)	*P* value	EHR (95% CI)	*P* value	EHR (95% CI)	*P* value
Sex (Ref=female)												
Male	1.06 (0.83–1.37)	0.62	1.06 (0.83–1.36)	0.64	0.74 (0.55–0.99)	0.041	0.75 (0.56–1.01)	0.059	1.51 (1.21–1.88)	<0.001	1.46 (1.17–1.82)	0.001
Age (Ref = <65 years)												
65–69 years	1.40 (1.00–1.96)	0.052	1.41 (1.01–1.98)	0.046	0.86 (0.56–1.33)	0.50	0.91 (0.59–1.41)	0.66	1.15 (0.85–1.54)	0.37	1.12 (0.83–1.50)	0.47
70–74 years	1.36 (0.96–1.93)	0.080	1.37 (0.97–1.94)	0.074	1.34 (0.86–2.08)	0.20	1.27 (0.81–1.99)	0.30	1.72 (1.28–2.32)	<0.001	1.68 (1.24–2.26)	0.001
75–79 years	1.81 (1.27–2.58)	0.001	1.79 (1.26–2.55)	0.001	1.37 (0.89–2.11)	0.149	1.41 (0.92–2.17)	0.115	2.32 (1.73–3.12)	<0.001	2.24 (1.66–3.02)	<0.001
≥80 years	2.74 (1.91–3.94)	<0.001	2.71 (1.88–3.89)	<0.001	2.70 (1.73–4.23)	<0.001	2.62 (1.67–4.10)	<0.001	3.33 (2.43–4.56)	<0.001	3.21 (2.34–4.41)	<0.001
Stage at diagnosis (Ref = localized)												
Regional to lymph nodes	52.86 (4.15–672.50)	0.002	42.14 (5.34–332.53)	<0.001	9.35 (1.57–55.73)	0.014	8.26 (1.71–39.97)	0.009	9.28 (5.23–16.45)	<0.001	9.48 (5.37–16.73)	<0.001
Regional by direct extension	118.50 (9.41–1491.84)	<0.001	94.30 (12.10–734.74)	<0.001	22.82 (3.90–133.61)	0.001	20.12 (4.24–95.46)	<0.001	12.13 (6.89–21.38)	<0.001	12.31 (7.02–21.58)	<0.001
Distant	389.21 (31.38–4826.74)	<0.001	310.41 (40.57–2375.25)	<0.001	90.48 (15.83–517.32)	<0.001	78.16 (16.93–360.80)	<0.001	23.47 (13.89–39.64)	<0.001	24.07 (14.31–40.48)	<0.001
Unknown	789.48 (56.42–11047.06)	<0.001	642.05 (72.85–5658.29)	<0.001	35.86 (5.74–223.80)	<0.001	27.94 (5.40–144.64)	<0.001	18.24 (8.96–37.12)	<0.001	18.67 (9.21–37.82)	<0.001
CCI score			1.08 (0.96–1.22)[a]	0.188			1.18 (1.02–1.37)[a]	0.025			1.13 (1.02–1.25)[a]	0.025

CCI, Charlson Comorbidity Index; CI, confidence interval; EHR, excess hazard ratio; Ref, reference.

All models include the baseline variables of sex, age at diagnosis, and cancer stage at diagnosis. Full models also include the CCI score in addition to the baseline variables.

[a] Expressed as the excess hazard ratio per one-point increase in CCI score.
